# Editorial: Medicinal plants from the Americas: a source of biologically active extracts and metabolites

**DOI:** 10.3389/fphar.2026.1956535

**Published:** 2026-09-08

**Authors:** Ana H. F. Castro, John T. Arnason, Irene Villasenor, Luciana A. Lima

**Affiliations:** 1 Universidade Federal de São João del-Rei, São João del-Rei, Brazil; 2 University of Ottawa, Ottawa, ON, Canada; 3 University of the Philippines Diliman DNA Analysis Laboratory, Quezon, Philippines

**Keywords:** Americas, ethnopharmacology, folk medicine, indigenous knowledge, medicinal plants

For millennia, medicinal plants from the Americas have anchored indigenous healing practices, offering natural remedies for a wide range of illnesses. In recent years, due to emerging diseases and antibiotic resistance, the growing demand for new therapeutic agents has led to increased scientific interest in therapeutic substances from plant biodiversity. By combining traditional knowledge with modern science, researchers are discovering the powerful potential hidden within the extracts and metabolites of plants. The primary objective of this topic was to foster interdisciplinary studies exploring the biologically active compounds found in medicinal plants from the Americas. By bringing together diverse areas of expertise, including ethnobotanists documenting traditional plant uses, chemists isolating and characterizing active compounds, and pharmacologists evaluating their therapeutic properties, this Research Topic aimed to build a comprehensive understanding of these botanical resources.

Mitsi and Echeverría reviewed archaeological, ethnohistorical, and ethnographic evidence supporting the ubiquitous use of fermented beverages in pre-Hispanic Chile made from a variety of plant-based raw materials. The review provided a comprehensive analysis of interdisciplinary information on traditional Chilean fermented beverages, as well as the raw materials used in their production, and highlighted the importance of integrating ethnopharmacological perspectives into modern research efforts.

Based on phytochemical, *in vitro* and *in silico* studies, Torres-Benítez et al. identified fourteen compounds in the aqueous extract of *Lophosoria quadripinnata*, a fern species from the Dicksoniaceae family, widely distributed in Central and South America. *In vitro* and *in silico* assays revealed that 5C3M (5-*O*-caffeoyl-3-*O*-malonylquinic acid), 5GDC (5-*O*-glucoside 6,7-dimethoxycoumarin), and irifloside possess significant potential for the development of neuroprotective agents, especially in diseases related to oxidative stress such as Alzheimer’s and Parkinson’s.

Aguirre et al. investigated the *in vitro* and *in silico* interactions of three chromene-type flavanones isolated from *Dalea boliviana* (Fabaceae) with xanthine oxidase (XO), a key enzyme involved in oxidative stress-related disorders. The study explored their potential as XO inhibitors and their possible therapeutic applications in diseases associated with hyperuricemia.

*Asparagus officinalis* (Asparagaceae) is a versatile, nutrient-dense, low-calorie vegetable that contains various bioactive metabolites that have shown a variety of biological functions beneficial to health. In their article, Fang et al. reported strong preclinical evidence of the potential therapeutic benefit of *A. officinalis* extract as a novel dietary strategy for the treatment of endometrial cancer.

Brache and Diazgranados reviewed plants traditionally used for neuropsychiatric disorders in Colombia, which has a diverse and intensively used medicinal flora. Their analysis of 42 species for psychoactive, stimulant, sedative, anxiolytic, and cognitive effects revealed that six species warrant further investigation. These species are *Lochroma fuchsioides, Brunfelsia grandiflora, Souroubea corallina, Tabernaemontana heterophylla, Psidium guajava, and Dianthera pectoralis*, which emerged as recurrently cited across ethnobotanical and pharmacological sources.

Carballo-Arce et al. reviewed the ethnobotanical, phytochemical and medicinal potential of the Marcgraviaceae, a tropical family of over 100 species of lianas and shrubs. Although relatively few species have been reported in the literature, available studies revealed promising activity. For example, *Marcgravia* spp., traditionally used for dermatological conditions, inhibited bacterial quorum sensing, with active principles identified as naphthoquinones. *Schwartzia brasiliensis* has demonstrated antiviral activity against dengue virus, *in vivo* antimalarial efficacy and anti-inflammatory properties, *Souroubea* spp. has demonstrated anxiolytic activity in animals with triterpenes identified as active principles.

Yang and Spagnuolo identified 6-methoxydihydroavicine (6ME), a benzophenanthridine alkaloid (BPA) derived from the genus *Macleaya* as a potent compound that reduced acute myeloid leukemia (AML) cell viability. Their findings demonstrated that 6ME exerts strong cytotoxic effects against AML cells through mitochondrial dysfunction and ROS-mediated apoptosis. They concluded that it has promise as a therapeutic agent.

A comprehensive review was carried out by Fuentes-Barros et al. on *Cryptocaraya alba*, an endemic Chilean tree traditionally used as a food source and for a variety of ethnomedicinal applications in both human and non-humans. Extensive phytochemical studies revealed the presence of polyphenols, terpenoids, and alkaloids. Biological, pharmacological, as well as toxicological, pre-clinical, and clinical studies on the plant are still limited.

In a review, Peralta et al. reported on the active principles responsible for the antifungal activity of *Dalea pazensis*, particularly that of the chloroform extract. Phytochemical characterization identified (2S)-5,7,2′,4′-tetrahydroxy-5′-(1‴,1‴-dimethylallyl)-8-prenylflavanone, a compound previously associated with antifungal activity and efflux pump inhibition, as the major constituent of this extract, and β caryophyllene as the main component of the essential oil. The results highlighted this species as a promising source of prenylated flavanones possessing dual antifungal and anti-tyrosinase activity, as well as bioactive volatile compounds with antifungal activity.

In conclusion, this focussed Research Topic of articles reported the biological and pharmacological activities of several medicinal plants of current interest from the Americas, offering novel data that warrant future investigations.

